# Refractory Hypotension in an 80-Year-Old Man: A Case Report

**DOI:** 10.7759/cureus.49424

**Published:** 2023-11-26

**Authors:** Amit Banerjee, Moe Wutye Kyaw

**Affiliations:** 1 Diabetes and Endocrinology, Royal Liverpool Hospital, Liverpool, GBR; 2 Internal Medicine, St Helens and Knowsley NHS Foundation Trust, Merseyside, GBR

**Keywords:** blood pressure, refractory shock, steroid use, low blood na+, adrenal disease

## Abstract

This case report presents the management and diagnosis of an 80-year-old gentleman who presented to the Accident and Emergency Department with refractory hypotension, hypoglycemia, and hyponatremia. Initially diagnosed with sepsis and cellulitis, it was later discovered that the patient was suffering from an Addisonian crisis due to adrenal insufficiency induced by long-term steroid use for rheumatoid arthritis. Prompt administration of intravenous hydrocortisone and fluid resuscitation led to a significant improvement in the patient's condition. This case emphasizes the importance of considering adrenal insufficiency in patients with similar clinical presentations and highlights the critical role of early diagnosis and treatment.

## Introduction

Addisonian crisis is a life-threatening condition resulting from adrenocortical insufficiency. It is often challenging to diagnose due to its nonspecific clinical presentation, which may include fatigue, weakness, nausea, vomiting, abdominal pain, back pain, diarrhea, dizziness, hypotension, syncope, and shock [1]. Usually, Addisonian crisis is not at the top of the differential compared to other more common diagnoses [1]. This case report illustrates the clinical course, diagnosis, and management of an elderly gentleman initially presenting with symptoms resembling sepsis, ultimately attributed to adrenal insufficiency and crisis induced by prolonged intermittent steroid use. Prompt recognition of the Addisonian crisis is essential to improve patient outcomes.

## Case presentation

An 80-year-old male patient, largely housebound due to frailty and poor mobility, was brought to the Accident and Emergency Department with a history of feeling unwell and lethargic for three days prior to the presentation to the hospital. He denied any history of cough, dysuria, headache, or joint pain. He noticed some redness over his left lower leg for the last five days, which is painless.

His past medical history included chronic kidney disease (CKD stage 3), heart failure (HF), type 2 diabetes mellitus (T2DM), and rheumatoid arthritis. He was taking ramipril 2.5 mg once daily, Bisoprolol 1.25 mg once daily, frusemide 40 mg once daily, and gliclazide 40 mg once daily. He was not on any regular treatment for rheumatoid arthritis.

Upon examination, the patient was diagnosed with cellulitis of the left leg due to a non-tender warm-to-touch erythematous lesion. His blood pressure was 60/40 mmHg, and his blood glucose level was 1.7 mmol/L. He was then suspected to be having sepsis due to an obvious source of infection (left leg cellulitis), low blood pressure, and raised lactate (3.7 mmol/L). The medical team started with prompt treatment with broad-spectrum intravenous antibiotics (as per the local antimicrobial policy). The medical team also prescribed an intravenous 0.9% sodium chloride bolus and corrected hypoglycemia with intravenous dextrose. Despite these measures, the patient's blood pressure remained critically low (60/40 mmHg), though blood glucose level improved to 5.3 mmol. At this point, the patient already had four and a half liters of Intravenous 0.9% sodium chloride for resuscitation. Table 1 presents the vital observations of the patient at the time of admission to the Accident and Emergency Department.

**Table 1:** Patient's vital observations

Parameter	Record
Blood pressure ( BP )	60/40 mmHg
Heart rate ( HR )	111 bpm
Temperature ( axillary )	35.6 °C
Oxygen saturation	96% on Room air
Respiratory rate	16 breaths per minute
Capillary blood glucose	1.7 mmol/L

A consultation with the Intensive Care Unit (ICU) concluded that the patient's frail health rendered him unsuitable for ICU (level 3) care. A ward-based approach was adopted, which meant that the patient would not be a candidate for intubation, ventilatory support, central venous line, or vasopressor support. A Do not Attempt Cardio-pulmonary Resuscitation Form (DNACPR) was signed. The medical team informed the family about the potential that the patient may not recover and may pass away during this admission. A decision was made that the patient would be treated in the monitored bed of the resuscitation unit of the Emergency Department and acute medical ward. Table 2 displays the laboratory results obtained during the patient's evaluation.

**Table 2:** Laboratory results

Test	Results	Normal range
Haemoglobin (Hb)	11.6 g/dL	13.8 – 17.2 g/dL
White cell count (WCC)	17,000 cells/mcL	4,500 – 11,000 cells/ mcL
Sodium (Na)	131 mmol/L	135-145 mmol/L
Potassium (K)	4.7 mmol/L	3.5-5.1 mmol/L
Urea	11.4 mg/dL	7-20 mg/dL
Creatinine	2.3	0.6-1.2 mg/dL
Bicarbonate (HCO3)	23 mmol/L	22-28 mmol/L
Random cortisol	6.1 mcg/dL	6.2-19.4 mcg/dL
Thyroid stimulating hormone (TSH)	2.1 mIU/L	0.4-4.0 mIU/L
Free T4	1.4 ng/dL	0.8 – 1.8 ng/dL
Lactic acid	3.7 mmol/L	0.5 – 2.2 mmol/L
Ph (in venous blood sample)	7.31	
Random venous blood glucose	2.8 mmol/L	3.9 – 7.8 mmol/L
CRP	44 mg/L	< 10 mg/L
Blood culture	Sent to lab	

Upon further reviewing of the patient's past clinical notes, it was found that the patient was prescribed Prednisolone four times (and gradually weaned over three to four weeks each time) in the last 12 months for his Rheumatoid arthritis by the patient's general practitioner. The patient had been referred to the Rheumatology team of his local hospital for consideration of steroid-sparing therapy, but the patient had not been seen yet in the rheumatology outpatient department due to a long waiting list. This crucial piece of information was not obtained in the initial brief history taking.

Recognizing the potential link between steroid use and the patient's presentation, 200 mg of hydrocortisone was administered intravenously with 500 ml of 0.9% NaCl (Sodium Chloride) bolus. Subsequently, the patient's blood pressure improved to 95/70 mmHg within 30 minutes. He was then transferred to the acute medical unit from the Emergency Department, where he experienced an uneventful recovery. He was initially treated with intravenous hydrocortisone 50 mg 6 hourly alongside Intravenous antibiotic and Intravenous fluid. Once the patient started to show signs of clinical recovery, IV antibiotic was switched to oral antibiotic. His IV hydrocortisone was switched to 20 mg oral Hydrocortisone twice daily for five days, followed by 10 mg twice daily to be continued.

Further investigation with a short Synacthen test confirmed the patient's adrenocortical insufficiency due to prolonged steroid use for rheumatoid arthritis. The test was performed when the patient’s acute illness was treated. The patient was referred to the Endocrine outpatient clinic for follow-up and long-term care. The patient was explained about the sick day rules which meant that the patient must double the dose of his oral hydrocortisone for a short period of time whenever he gets sick. An emergency rescue injection pack for IM Hydrocortisone was provided with the discharge medications in case the patient is unable to take hydrocortisone orally due to any reason (e.g., vomiting).

## Discussion

The adrenal cortex secretes the essential steroid hormones, cortisol and aldosterone, under the control of pituitary adrenocorticotropic hormone (ACTH), angiotensin II, and plasma potassium. Exogenous steroid use is the most frequent cause of adrenal insufficiency [2]. Up to 2.5% of the population is taking such steroid medications for inflammatory or immune-mediated conditions [3]. Exogenous steroid causes suppression of ACTH, and in case of chronic suppression of ACTH, cortisol produces part of the adrenal cortex (zona fasciculata and zona reticularis) atrophies. These individuals are vulnerable to developing adrenal crisis if the steroid is stopped abruptly. They are also at higher risk of developing adrenal crisis in case of physical stress (infection, dehydration, trauma, or surgery) or emotional stress if the dose of steroid is not increased significantly [4].

In this clinical case, hypoglycemia was deemed secondary to gliclazide (an oral hypoglycemic agent that the patient was taking for his type 2 diabetes), and low blood pressure was deemed due to sepsis. A thorough past medical history was not obtained due to critical clinical presentation, where attention was diverted to stabilizing vital parameters (low BP and blood glucose level). As a result, the diagnosis of a potentially life-threatening cause was delayed. It highlights the importance of proper history taking in reaching a correct diagnosis, even in case of emergency. It is important to remember that a short course of treatment with steroids is never harmful (including pregnancy) [5]. So, if in any doubt regarding the diagnosis of adrenal crisis, prompt treatment should always be administered. In this patient, we deem that the adrenal gland was compromised due to prolonged intermittent use of prednisolone, and an adrenal crisis was triggered due to the concomitant infection (lt leg cellulitis).

This case underscores the significance of considering adrenal insufficiency in patients with hypoglycemia, hyponatremia, and refractory hypotension, particularly when a history of prolonged steroid use is present. Addisonian crisis, although rare, requires immediate treatment with intravenous hydrocortisone to prevent potentially fatal outcomes. Timely recognition and treatment can lead to rapid improvement in the patient's condition, as demonstrated in this case.

## Conclusions

This case report highlights the critical importance of recognizing and promptly managing Addisonian crisis in patients with hypoglycemia, hyponatremia, and refractory hypotension, especially in those with a history of prolonged steroid use. Early administration of intravenous hydrocortisone is essential for favorable patient outcomes. Physicians should maintain a high index of suspicion for adrenal insufficiency in such clinical scenarios.
